# Expression Profiling of Attenuated Mitochondrial Function Identifies Retrograde Signals in *Drosophila*

**DOI:** 10.1534/g3.112.002584

**Published:** 2012-08-01

**Authors:** William A. Freije, Sudip Mandal, Utpal Banerjee

**Affiliations:** * Department of Molecular, Cell, and Developmental Biology, University of California, Los Angeles, California 90095; † Molecular Biology Institute, University of California, Los Angeles, California 90095, and; ‡ Eli and Edythe Broad Center of Regenerative Medicine and Stem Cell Research, University of California, Los Angeles, California 90095

**Keywords:** electron transport, glycolysis, cytochrome oxidase Va, lactate, microarray

## Abstract

Mitochondria are able to modulate cell state and fate during normal and pathophysiologic conditions through a nuclear-mediated mechanism collectively termed as a retrograde response. Our previous studies in *Drosophila melanogaster* have clearly established that progress through the cell cycle is precisely regulated by the intrinsic activity of the mitochondrion by specific signaling cascades mounted by the cell. As a means to further our understanding of how mitochondrial energy status affects nuclear control of basic cell decisions, we have employed Affymetrix microarray-based transcriptional profiling of *Drosophila* S2 cells knocked down for the gene encoding subunit Va of the complex IV of the mitochondrial electron transport chain. The profiling data identify transcriptional upregulation of glycolytic genes, and metabolic studies confirm this increase in glycolysis. The data provide a model of the shift of metabolism from a predominately oxidative state toward a predominately aerobic glycolytic state mediated through transcriptional control. The transcriptional changes alter many signaling systems, including p53, insulin, hypoxia-induced factor α, and conserved mitochondrial retrograde responses. This rich dataset provides many novel targets for further understanding the mechanism whereby the mitochondrion manages energy substrate disposition and directs cellular fate decisions.

Mitochondria are dynamic cellular organelles that act as metabolic hubs to integrate diverse cell extrinsic and intrinsic signals that modulate cell proliferation, differentiation, and death (DiMauro and Schon 2003; Ott *et al.* 2009; Mandal *et al.* 2011;). The *Drosophila* mitochondrial genome encodes only 13 proteins (Garesse and Kaguni 2005), with the vast majority of proteins involved in mitochondrial structure and function encoded by the nuclear genome. Therefore, proper communication between mitochondria and the nucleus is essential for maintaining cellular homeostasis. As mitochondrial biogenesis is completely dependent on the nuclear genome, much attention has been paid to understanding anterograde regulation, the mechanism by which information and materials are transferred from the nucleus and cytoplasm to the mitochondria. However, recent studies in diverse organisms have uncovered a unique process of retrograde regulation by which mitochondria exert specific effects on nuclear function and thereby modulate cellular function under normal and pathophysiological conditions. Although the phenomenon of mitochondrial retrograde regulation is conserved from yeast to humans, the molecular mechanisms underlying the process vary across phyla (Butow and Avadhani 2004).

In budding yeast, the organism most investigated for mitochondrial retrograde signaling, a group of transcription factors known as retrograde (RTG) proteins are involved in transducing a mitochondrial dysfunction signal to the nucleus (Liu and Butow 2006). Through intranuclear translocation, the RTG proteins induce the transcription of specific target genes, which in turn modulate mitochondrial function. A primary function of the RTG target genes is to maintain glutamate supplies to meet biosynthetic needs, as glutamate through the amine derivative glutamine provides all the nitrogen used in biosynthetic reactions. Retrograde regulation in higher plants, as observed in *Brassica juncea*, involves the MAPK signaling pathway in modulating the expression of nuclear genes associated with cytoplasmic male sterility (Yang *et al.* 2008). Signaling from mitochondria to nucleus has also been evidenced in mammalian cells. Using C2C12 skeletal myoblasts, mitochondrial stress was found to increase intracytoplasmic calcium ion levels and subsequently activate calcineurin (Biswas *et al.* 1999). In a model of cancer, osteosarcoma cells depleted of mitochondria were observed to have increased *inosine 5′-monophosphate dehydrogenase type 2* and *ubiquinol cytochrome-c reductase core protein I* proteins as a response to mitochondrial depletion (Kulawiec *et al.* 2006). This increase in protein production was returned to baseline wild-type levels with repletion of mitochondria through cybrid formation, indicating continuous monitoring and a reversible feedback control.

In recent years, our studies with the genetically tractable organism *Drosophila melanogaster* led to the identification of two independent retrograde signaling pathways that are activated upon mitochondrial dysfunction and impose a block in G1–S progression during the cell cycle (Mandal *et al.* 2005; Liao *et al.* 2006). Molecular genetics analyses revealed that cells mutant for the gene encoding *Cytochrome c oxidase subunit Va* (*CoVa* ) of complex IV of the electron transport chain specifically activate a retrograde signaling pathway that involves both *AMP-activated protein kinase* and *p53*. The activated *p53* leads to transcriptional activation of *archipelago*, the F-box protein responsible for specific ubiquitinylation of *CyclinE* (Mandal *et al.* 2010). The targeting of *CyclinE* results in proteasomal degradation and thereby imposes a block in G1–S progression. Interestingly, despite a significant drop in cellular ATP level, the *CoVa* mutant cells do not apoptose, undergo normal differentiation, and can even send axonal projections to the brain. This suggests that apart from activating a G1 cell-cycle checkpoint, retrograde signaling in *CoVa* mutant cells also modulates nuclear gene expression to support cell survival and activity in an altered metabolic condition. To better understand the genome-wide response to mitochondrial dysfunction, we have employed Affymetrix 3′ gene expression microarrays to define the transcriptional changes in *Drosophila* S2 cells knocked down for *CoVa* as a follow-up of our initial mechanistic studies.

The transcriptional profiling experiments described herein reveal that with loss of *CoVa* by RNA interference (RNAi) there is upregulation of glycolytic genes, thereby demonstrating a shift from oxidative phosphorylation to aerobic glycolysis. A systems biologic interpretation of the most highly differentially expressed genes reveals a portrait of the cellular response to abrogation of electron transport function and identifies the specific genes the cell uses to acquire glucose, control the metabolism through the glycolytic pathway, and generate and dispose of metabolites. Conserved signaling pathways are also found within this data. These responses include the action of p53, insulin, hypoxia-induced factor α (*Hifα*), stress oxidant responses, and other conserved mitochondrial retrograde signals. This transcriptional data therefore supplies important models of cell-cycle control, energy management, and conserved mitochondrial retrograde responses.

## Materials and Methods

### *CoVa* RNA interference in S2 cells and microarray expression profiling

RNAi using a sequence specific to *CoVa* was performed in *Drosophila* S2 cells as previously described (Mandal *et al.* 2005). A GFP sequence not found in the *Drosophila* genome was used as an experimental control. A DNA template for *in vitro* transcription was amplified using *CoVa* primer sequences TAATACGACTCACTATAGGCTGCTACTCGTAA (forward) and TAATACGACTCACTATAGGGTACTTCGTA (reverse); GFP primer sequences TAATACGACTCACTATAGGGAGTGAA (forward) and TAATACGACTCACTATAGGGAGCTTC (reverse). A plasmid containing the GFP coding sequence was kindly provided by Dr. Arnold Berk of the Molecular Biology Institute at UCLA. Using the DNA template amplified from either *CoVa* or GFP, interfering RNA specific for *CoVa* and GFP were *in vitro* transcribed using Megascript T7 RNA polymerase (Ambion, Austin, TX). Cultured *Drosophila* S2 cells were transfected with 20 μg of RNA specific for either GFP or *CoVa* using calcium phosphate. In the pilot experiment, cells were harvested at 48 and 168 hr after transfection. In the second and third replicates, cells were harvested at 72, 96, 120, and 168 hr after transfection for a total of three independent time course experiments. At the time of cell collection, low-speed centrifugation and washing in PBS was performed. Total RNA was extracted using trizol (Invitrogen; Carlsbad, CA) as per the manufacturer's protocol. RNA cleanup using the mini RNEASY column was performed as per the manufacturer's protocol (Qiagen; Valencia, CA). RNA quality was ensured by spectrophotometric absorption at 260nm/280nm, as well as by the Agilent Bioanalyzer, which ensured integrity of the small and large ribosomal subunits and lack of degradation (Agilent; Santa Clara, CA).

Total RNA (1 μg) was used to generate microarray probes by standard Affymetrix protocol (Enzo Diagnostics; Farmingdale, NY), which were hybridized to the Affymetrix *Drosophila* genome 2 arrays (Affymetrix; Santa Clara, CA). The Gene Chip Operating System was used to define absent/present calls and generate cel files using the default settings. Data files (cel) and corresponding text files were uploaded into the dCHIP program and normalized to the median intensity array. Pairwise comparisons were made between the GFP controls and the *CoVa* RNA–interfered S2 cells.

### Quantitative reverse-transcription polymerase chain reaction

*Phosphofructokinase* (*Pfk*), *phosphoglycerate kinase* (*Pgk*), and the *Drosophila* homolog of lactate dehydrogenase, *Ecdysone-inducible gene L3* (*Impl3*), were evaluated by quantitative reverse-transcription polymerase chain reaction (qRT-PCR). RNA (1 μg) from the 72 hr time point was evaluated by Super Script Platinum SYBR green One Step q-PCR according to manufacturer's protocol (Invitrogen) using an ABI 7500 thermocycler with the following parameters: 50 C for 10 min, 95 C for 5 min, then 40 cycles of 95 C for 15 sec and 60 C for 60 sec. Data were normalized to *Rpl10*, and relative quantification to the GFP sample was made using the ΔΔCt method (Livak and Schmittgen 2001). *Rpl10* was used as an amplification control and was selected from a survey of the microarray data for genes with the lowest coefficient of variation. The sequences of the primers are as follows: *Impl3* forward, ATGGCATTGACAAGGATGTG; *Impl3* reverse, GACATGATGTTGGCGGACTT; *Pfk* forward, AGACGATGGGTGGCTACTGT; *Pfk* reverse, GGCCATGTGGTAGACATCCT; *Pgk* forward, AATTGTCGCTGCCTTGGATA; *Pgk* reverse, GGTGCCAGGGTGTACTTGAT; *Rpl10* forward, AAGAAGGTGCTCTGCCTGTC; *Rpl10* reverse, CGCACATTCTGCCAGTTCT. Statistical significance was evaluated by Fisher's protected least significant difference using StatView software version 5 (SAS, Cary, NC).

### Lactate measurement

The conditioned media from the S2 cells was collected at 168 hr after either GFP (control) or *CoVa* RNAi. Lactate measurements were made in triplicate using an enzyme-linked ultraviolet absorption method (Raisio Diagnostics, Rome, Italy) calibrated to known standards. Statistical significance was evaluated by Fisher's protected least significant difference.

### Quantification of glycolysis through the extracellular acidification rate of S2 cell cultures

S2 cells were cultured in Schneider's media, and *CoVa* and GFP knockdown was performed using small interfering RNA as previously described (Mandal *et al.* 2005). Cells were subcultured every 3 days, and daily cell counts were assayed. One hundred thirty-two hours after RNAi treatment, coincident with the growth plateau seen in *CoVa* knockdown cells and with the observed shift to glycolytic gene expression found in the microarray analysis, the cells were counted and seeded into the proprietary Seahorse culture plate for a projected density of 50,000 cells per milliliter after an overnight rest. The following morning (144 hr), the cell media was decanted and replaced with DMEM low-glucose media (glucose 2.5 mM) for 5 hr. Real-time measurements of the media pH was made as an index of the cellular response to a glucose load (25 mM), followed by a bolus of 2-deoxyglucose (2dG, 225 mM) as a specific inhibitor of glycolysis in the extracellular flux (XF) instrument (Seahorse Bioscience; Chicopee, MA). The resulting graph of extracellular pH over time indicates the glycolytic capacity of the cells, and using the area under this curve (AUC), direct comparison of the GFP controls with the *CoVa* knockdown cells was made after correcting for cell count, which was performed immediately after the extracellular flux measurements were recorded. The experiment was performed independently three times, and statistical significance for each experiment was evaluated by *t*-test.

## Results

**Figure 1:**
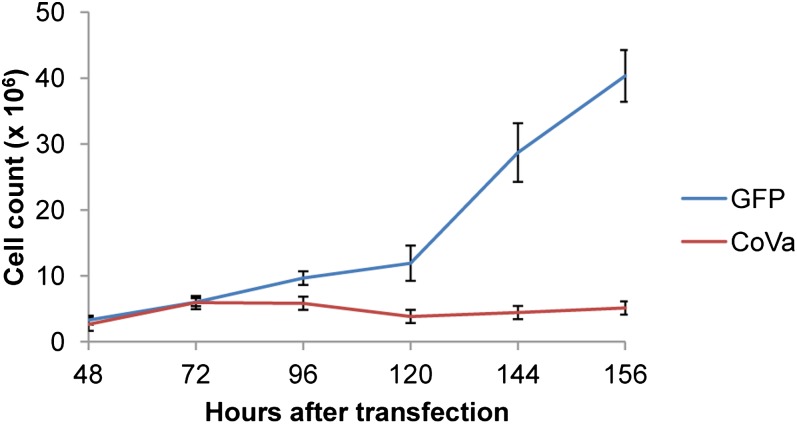
Proliferation profile of *Drosophila* S2 cells treated with either GFP or *CoVa* RNAi. S2 cells were treated with either GFP (control, blue) or *CoVa* interfering RNA (red). After initial rounds of mitosis, *CoVa* RNAi–treated cells slow down and stop dividing. Error bars indicate standard deviation.

Growth of *Drosophila* S2 cells is slowed when RNAi is used to abolish *CoVa* transcription (Figure 1), consistent with both *in vivo* and *in vitro* prior studies from our laboratory (Mandal *et al.* 2005). S2 cells transfected with a GFP control retain proliferative capacity after transfection, whereas *CoVa* knockdown cells display slowed growth by 96 hr after transfection. Using these growth kinetics, we selected 72, 96, 120, and 168 hr as time points to perform microarray expression profiling. Microarray signal intensity of *CoVa* transcripts revealed a knockdown that paralleled the cell growth kinetics, with a mean knockdown of 70% at the termination of the experiment (supporting information, Figure S1). The expression profile time series was performed in triplicate and is available at GEO accession number GSE32912.

### Transcriptional profiling identifies groups of genes that are coordinately and consistently altered by loss of electron transport function through *CoVa* RNAi

**Figure 2:**
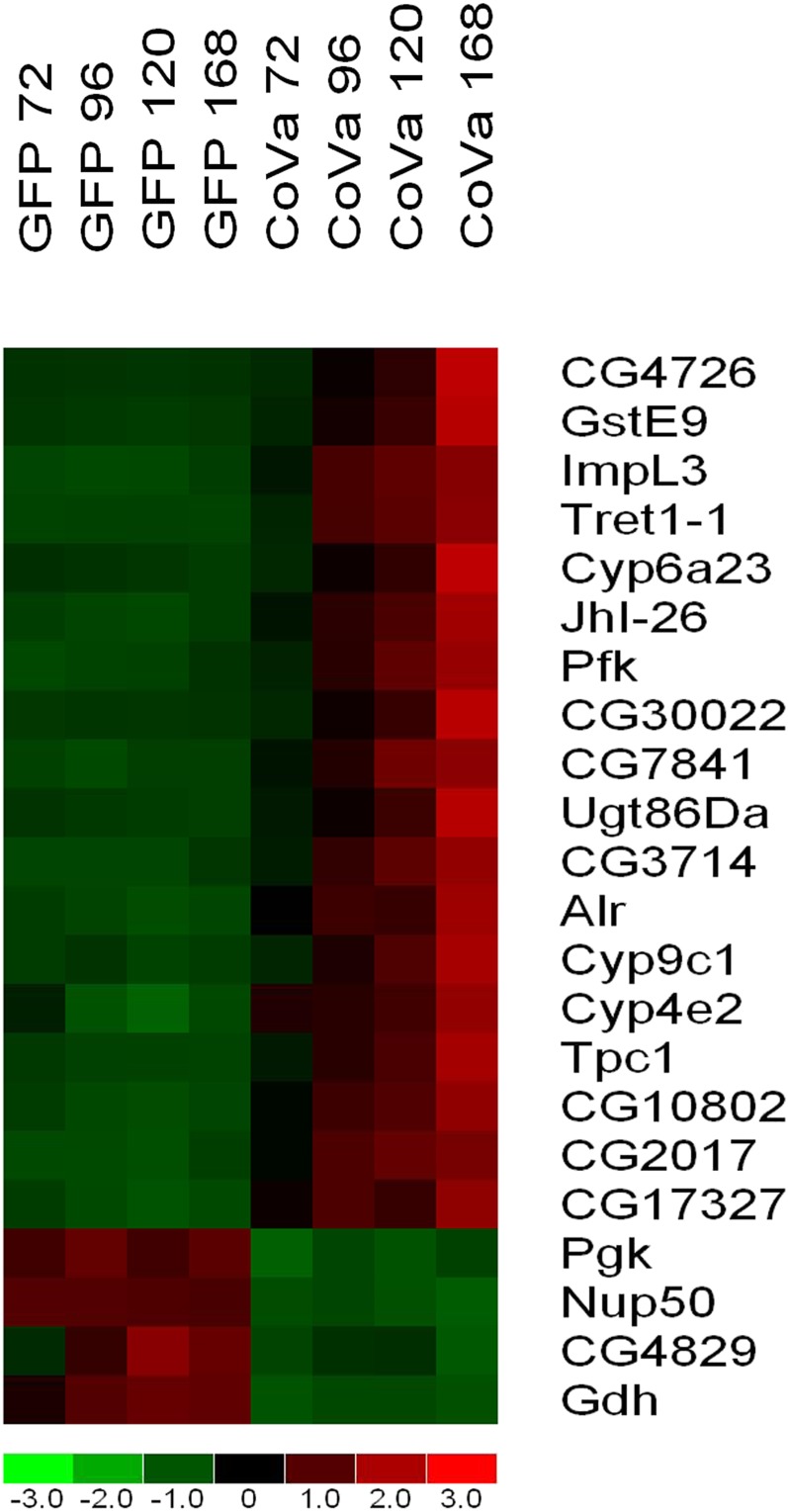
Microarray expression profiling identifies genes differentially expressed by *CoVa* knockdown in *Drosophila* S2 cells. Using microarray-based transcriptional profiling, 22 genes are identified to be differentially expressed as a result of *CoVa* knockdown in *Drosophila* S2 cells (see text for comparison criteria). Listed from left to right are the time-course samples of S2 cells with GFP indicating control and *CoVa* indicating *CoVa* knockdown. The number following the identifier is the number of hours after RNA interference was initiated. Listed from top to bottom are genes most highly upregulated to most highly downregulated. The color map bar on the bottom of the figure displays fold change of the gene expression, with red indicating a fold change of 1 or more, and green indicating a fold change of −1 or less. For a description of the specific genes, see the *Results* and *Discussion* sections.

To identify the genes most reliably changed by loss of *CoVa*, a stringent pairwise comparison was made between all control time points and all *CoVa* time points using all three independent time course experiments and the following comparison criteria: a minimum of 2-fold or greater difference below a 90% confidence bound; absolute difference greater than 500 (which is greater than 5-fold higher than the noise floor); and a *P* value less than 0.05 using a Welch-modified two-sample *t*-test. This identified 25 probesets consisting of 22 genes consistently and robustly altered by loss of *CoVa* expression (Figure 2). Of these 22 significantly and differentially expressed genes, 18 are upregulated and 4 are downregulated. To identify the effect of time in culture, a repeated measures analysis of variance was performed. This analysis identified that 19 of these 22 genes are statistically significantly different, indicating that time in culture after initial knockdown was related to the degree of expression changes (*P* range from 1 × 10^−5^ to 0.03). Three genes, Tret1-1, Nup50, and CG4829, were not found to be significantly different using a repeated measures analysis of variance, indicating that the abrupt, profound, and unwavering changes in their gene expression are not correlated to time in culture after initial knockdown. Despite the lack of significance by the repeated measures test, the expressions of these three genes are exquisitely correlated to *CoVa* knockdown and serve as time-independent markers of electron transport function. A less stringent comparison was made using the following criteria: a minimum of 1.5 times or greater difference below a 90% confidence bound; absolute difference greater than 200; and a *P* value less than 0.05 using a Welch-modified two-sample *t*-test (see Table S1). This less stringent comparison identified 142 probesets to be differentially expressed, with 111 probesets upregulated and 31 probesets downregulated.

### Glycolysis gene expression is strongly upregulated in response to *CoVa* RNAi

**Figure 3:**
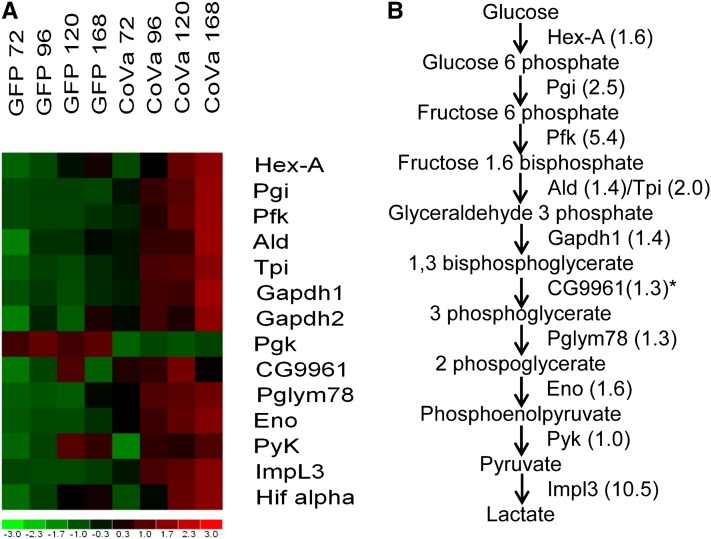
Glycolytic gene expression is increased as a response to *CoVa* knockdown in *Drosophila* S2 cells. The microarray data were collated for glycolytic gene expression. (A) The heat map of glycolytic genes. (B) The glycolysis pathway, enzymes, and fold changes at 168 hr after *CoVa* knockdown. The heat map displays from top to bottom the glycolytic genes arranged by their position in the glycolytic pathway, with the gene at the bottom of the figure Hifα, a transcription factor known to transcriptionally control glycolytic gene expression. Listed from left to right are the time course samples of S2 cells, with GFP indicating control and *CoVa* indicating *CoVa* knockdown. The number following the identifier is the number of hours after RNA interference was initiated. The color map bar on the bottom of the figure displays fold change of the gene expression, with red indicating a fold change of 1 or more, and green indicating a fold change of −1 or less. The specific genes from top to bottom are *Hexokinase-A* (*Hex-A*), *Phosphoglucose isomerase* (*Pgi*), *Pfk*, *Aldolase* (*Ald*), *Triose phosphate isomerase* (*Tpi*), *Glyceraldehyde 3 phosphate dehydrogenase 1* (*Gapdh1*), *Glyceraldehyde 3 phosphate dehydrogenase 2* (*Gapdh2*), *Pgk*, CG9961, *Phosphoglyceromutase* (*Pglym78*), *Enolase* (*Eno*), *Pyruvate kinase* (*PyK*), *Impl3*, and *Hifα*. ^*^The fold change of CG9961, the alternate *Pgk* equivalent that is increased by *CoVa* knockdown, is displayed.

The majority of genes identified in the stringent comparison are novel targets, as most are uncharacterized or possess a CG identifier. The 22 stringently identified genes were evaluated with the online software *Database for Annotation*, *Visualization and Integrated Discovery* of the National Institutes of Allergy and Infectious Diseases; this software identifies groups of genes that are overrepresented using an unbiased algorithm based upon the Gene Ontology classification (Huang da *et al.* 2009). This analysis identified glycolysis genes to be significantly overrepresented with loss of *CoVa* (software settings: functional annotation, gene list, AFFYMETRIX_3PRIME_IVT_ID, genelist). Overrepresentation of glycolysis genes was found using either the complete *Drosophila* genome as a background list (all 18,769 probesets found on the Affymetrix *Drosophila* Genome 2 microarray; *P* < 0.0007) or using an abbreviated background list containing probesets found to have a Present call in 100% of the samples (6670 probe sets; *P* < 0.03). We therefore examined the expression profile of all enzymes of the glycolytic pathway from hexokinase to lactate dehydrogenase (Figure 3). Although lactate dehydrogenase (*Impl3*) is not classically considered a member of the glycolysis pathway, it is required to dispose of pyruvate and allow continued metabolic substrate flux through glycolysis, particularly when disposal of pyruvate through the citric acid cycle and oxidative phosphorylation is impaired. Glycolytic genes are upregulated in response to *CoVa* knockdown. Interestingly, the described *Drosophila Pgk* is highly downregulated. Using an *in silico* approach, the *Drosophila* genome was surveyed for sequences similar to *Pgk*, and CG9961 was identified to have a high sequence similarity to *Pgk*. *Pgk* and CG9961 are located in tandem on *Drosophila* chromosome 2L. The transcripts of *Pgk* and CG9961 share 67% of their nucleotide sequence and 63% of their amino acid sequence. When similar amino acid residues are accounted for, the two proteins share 76% similarity using the basic local alignment search tool (BLAST) for protein pairwise comparison (Altschul *et al.* 1990). The microarray data reveals that when *CoVa* expression is downregulated, the expression of CG9961 is upregulated. Henceforth, we refer to CG9961 as *Drosophila* phosphoglycerate kinase 2 (*dPGK2*).

Upregulation of glycolytic genes prompted us to examine the expression of Hifα, the transcription factor known to be responsible for controlling glycolytic gene transcription (Figure 3)(Semenza *et al.* 1994). The microarray signal intensity of Hifα is increased by a mean of 50% as a response to *CoVa* knockdown, with a graded increase proportional to the time in culture after initial knockdown. When the microarray signal intensities of all of the GFP controls are compared with all *CoVa* samples there is a nonsignificant statistical trend toward increasing Hifα expression in the *CoVa* knockdown cells (*P* < 0.06). This difference peaks at the 168 hr time point, with a 70–90% increase in Hifα expression (*P* < 0.02).

### Glycolytic capacity of S2 cells is greatly increased by *CoVa* RNAi

**Figure 4:**
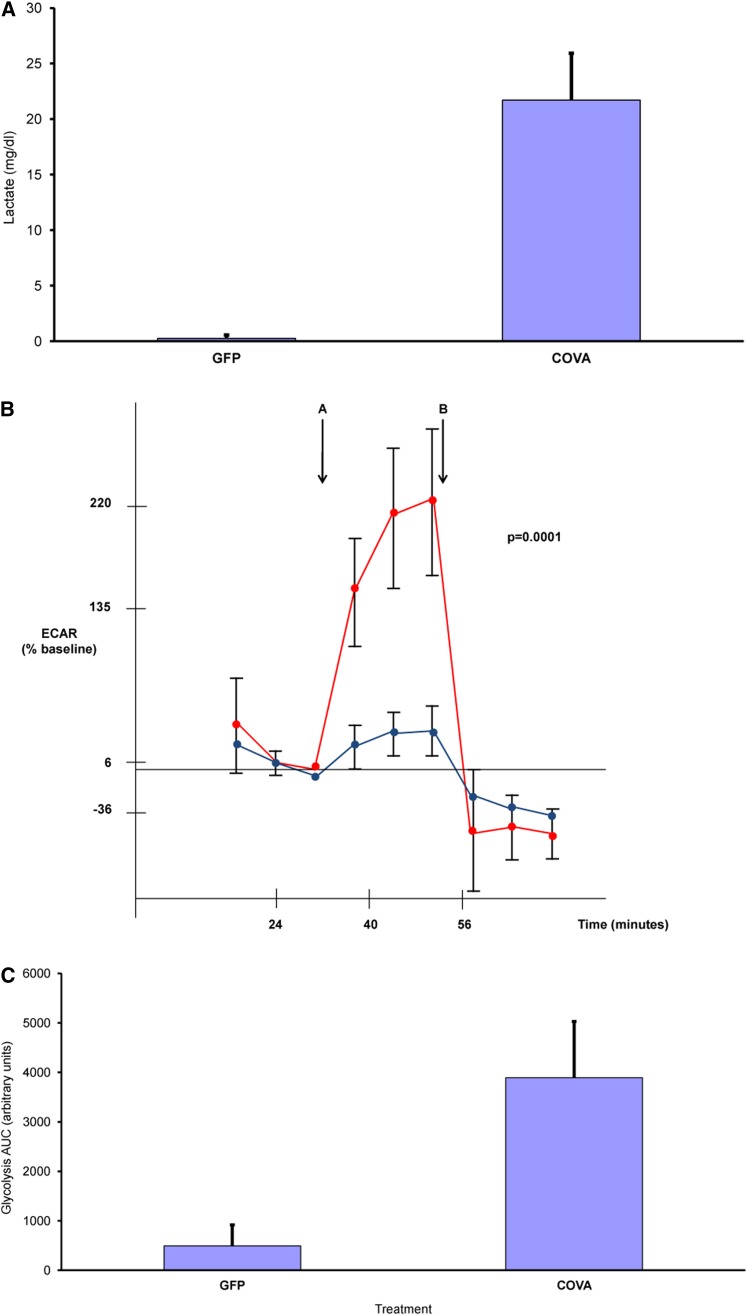
Abrogation of mitochondrial electron transport function through *CoVa* knockdown results in increased glycolysis and lactate production in *Drosophila* S2 cells. (A) Media concentrations of lactate are increased in response to *CoVa* knockdown in *Drosophila* S2 cells (*P* < 0.009). (B) Glycolysis rates are increased in *Drosophila* S2 cells when *CoVa* expression is knocked down. Media pH measurements were recorded in response to a bolus of glucose (point A) through administration of 2dG, a specific inhibitor of glycolysis (point B). The AUC is proportional to the cumulative glycolysis occurring in the cells. GFP controls are in blue and *CoVa* downregulated cells are in red. The extracellular acidification profile shown is from one of three independently performed experiments. (C) *CoVa* downregulated cells metabolize glucose through glycolysis at a rate 7.9 times the GFP controls (*P* < 0.0001).

To confirm the microarray results and evaluate the glycolytic capacity of *CoVa* knockdown cells, three additional confirmatory experiments were performed. First, qRT-PCR for selected glycolysis genes confirmed the microarray results (Table S2). qRT-PCR for *Pfk*, *Pgk*, and *Impl3* revealed significant differences between GFP controls and *CoVa* knockdown, and it paralleled the microarray results. The qRT-PCR results correlate to the microarray findings with a correlation coefficient of 0.9. Second, as aerobic glycolysis should result in increased lactate production, the lactate content of the conditioned S2 cell media would be an indirect assay for the glycolytic capacity of the cells. A lactate-specific spectrophotometric assay showed that the lactate concentration increased upon *CoVa* knockdown (Figure 4A). The conditioned S2 media concentration of lactate was 0.3 mg/dl in the GFP controls compared with 21.7 mg/dl in the *CoVa* knockdown (*P* < 0.009). Third, to conclusively assay the glycolytic capacity of the S2 cell cultures, metabolic flux studies using the Seahorse XF24 multiparameter analyzer was performed using glucose and 2dG as a specific inhibitor of glycolysis with real-time measurement of the pH of the extracellular media.

Cell culture of S2 cells was performed with *CoVa* knockdown as per the same protocol as the microarray experiments. At 144 hr after transfection, the metabolic flux studies were performed after placement of the cells in a 5 hr treatment of low glucose-containing medium (Figure 4B). When the glucose-limited cells were exposed to a bolus of glucose (arrow A), there was increased extracellular acidification found in the *CoVa* knockdown cells compared with the GFP controls. As the *CoVa* knockdown cells acidified the extracellular media to a greater extent, this suggests increased lactate production through increased glycolysis, with the end product of lactate being transferred to the extracellular space in *CoVa*-deficient cells. To specifically abrogate glycolysis, 2dG was then applied to the cells (arrow B). 2dG is an analog of glucose that is unable to undergo glycolysis. 2dG stopped the extracellular acidification of both the GFP controls and *CoVa*, but to a much larger degree in the *CoVa* knockdown cells. The AUC of the extracellular acidification rate from the time point of glucose injection through the 2dG treatment is proportional to the level of glycolysis occurring in the cells. *CoVa*-deficient cells had a 7.9-fold increase in the glycolysis AUC found compared with the *CoVa* knockdown cells (*P* < 0.0001)(Figure 4C). We conclude from these experiments that glycolysis is increased in response to abrogation of complex IV function through *CoVa* RNAi.

## Discussion

We have developed a rich dataset describing the transcriptional changes that are associated with RNAi-induced gene knockdown of *CoVa* in *Drosophila* S2 cells using microarray-based transcriptional profiling. The selected time points were designed to capture the cell-cycle arrest induced by loss of activity of the fourth complex of the electron transport chain to illuminate the mechanisms operative behind retrograde mitochondrial signaling and cell growth slowing (Mandal *et al.* 2005). The most striking aspect of these data is the transcriptional upregulation of glycolytic genes, which results in increased glycolytic capacity as confirmed in metabolic studies (Figures 3 and 4, respectively). Further scrutiny of the differentially expressed gene list clearly demonstrates the mechanism by which the cell directs energy disposition and controls cellular function.

### A systems biologic analysis outlines the specific genes responsible for energy substrate management in time of stress

**Figure 5:**
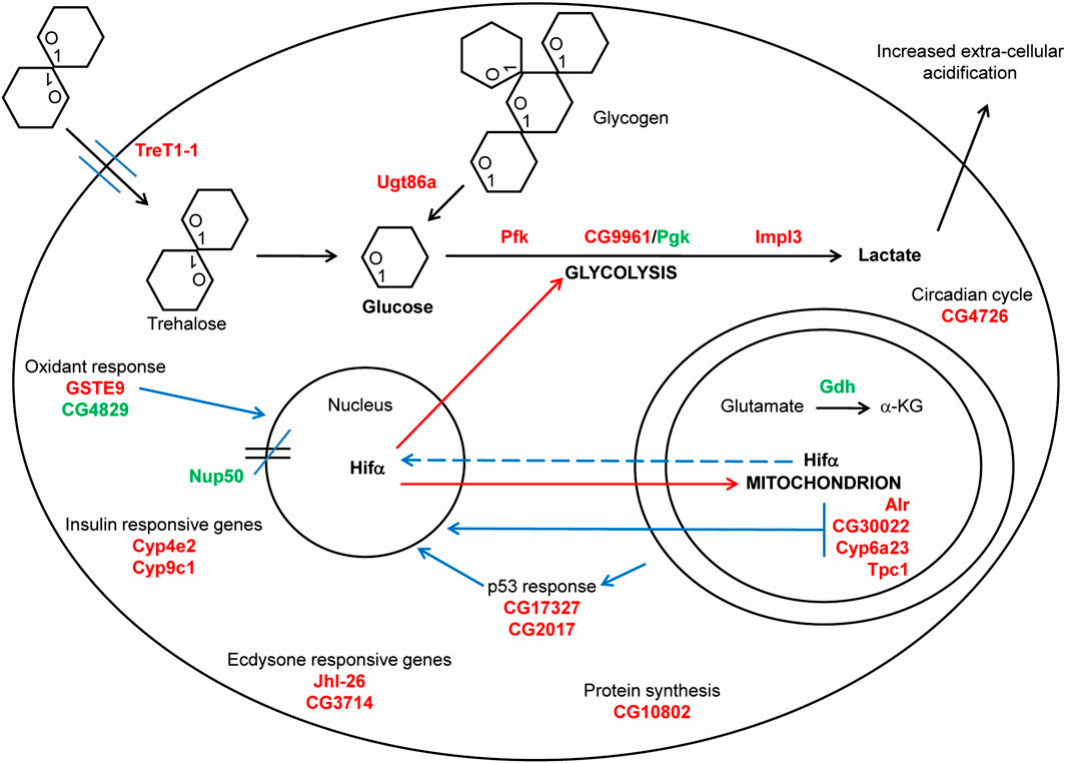
The 21 genes most differentially expressed in response to *CoVa* knockdown in *Drosophila* S2 cells identifies metabolic pathways and mitochondrial retrograde signals. After knockdown of *CoVa*, energy substrate disposition is dominated by the acquisition of glucose from both extracellular sources, such as from trehalose, and from intracellular sources, such as glycogen. The glucose is then metabolized through glycolysis and results in increased lactate production and the direction of reducing equivalents away from the mitochondrion by downregulation of glutamate dehydrogenase. The cellular response to electron transport deficiency also leads to the induction of specific mitochondrial retrograde signals. These signals include experimentally validated targets, including the mitochondrial proteins *Alr* and CG30022, as well as evidence of a p53-response, also experimentally validated, through the upregulation of the genes CG17327 and CG2017. *Hifα* provides a biologically relevant mitochondrial retrograde signal, as it is known to continuously sense ambient oxygen tension, the primary electron acceptor of the oxidative function of the mitochondrion. In addition, it is known to control glycolytic and cytochrome gene expression. Other pathways identified in the transcriptional response to *CoVa* knockdown include the ecdysone response, circadian cycling, new protein synthesis, and the closing of nuclear pores by affecting the importin-alpha and importin-beta nuclear pore complex. For a discussion of the specific genes, see the *Results* and *Discussion* sections. Genes in red indicate an upregulated gene, and genes in green indicate a downregulated gene. Solid black lines indicate a metabolic pathway, solid blue lines indicate an experimentally validated mitochondrial retrograde signal, dashed blue lines indicate a suspected mitochondrial retrograde signal, and red lines indicate genes whose expression is known to be controlled by Hifα (Semenza *et al.* 1994).

A systems biologic analysis of the 21 highest differentially expressed genes describes a dominant metabolic pathway defined by the acquisition of glucose from both extracellular and intracellular stores, utilization of the obtained glucose through the glycolytic pathway, and disposal of reducing equivalents to lactate production (Figure 5). This analysis contains 21 of the 22 genes identified and has excluded CG7841 as it is completely uncharacterized.

Trehalose, a disaccharide of glucose molecules linked at the first carbon position, is the primary circulating carbon source in insects. *Trehalose transporter 1-1* (*Tret1-1*) is highly upregulated in response to *CoVa* knockdown, thereby providing a mechanism by which trehalose may be drawn into the cell. In addition, *Ugt86a*, a UDP-glycosyl transferase with possible glycogen phosphorylase activity, may be able to mobilize glucose from intracellular glycogen stores, further providing glucose for glycolysis. The key glycolytic enzymes found to be upregulated by *CoVa* knockdown include *Pfk* and the *Drosophila* lactate dehydrogenase *Impl3*. The expression profiling also identifies a novel transcript, *dPGK2*, which encodes an alternative stress-induced enzyme containing homology to *Pgk* and serves the activity of *Pgk* during abrogation of electron transport function. Early attempts at characterizing the *Drosophila Pgk* identified at least three different electrophoretic variants (Chew and Cooper 1973), and subsequent studies using RNase protection assay and primer extension found multiple transcript clusters (Roselli-Rehfuss *et al.* 1992). Multiple isoforms of *Pgk* are found in *Drosophila*, indicating a regulatory mechanism similar to mammals. Future directed studies will clarify the role of *dPgk2* in times of mitochondrial stress.

Disposition of metabolic substrate to the glycolytic pathway is coordinately linked to substrate disposition away from the mitochondrion by modulating the production of the key anaplerotic intermediate α-ketoglutarate. There is likely decreased α-ketoglutarate production in the mitochondrion as the expression of *glutamate dehydrogenase* (*Gdh*), a primary mechanism to produce α-ketoglutarate in the mitochondrion, is rapidly and profoundly decreased. We therefore conclude that the electron transport deficiency caused by *CoVa* RNAi leads to a transcriptional response characterized by the preferential utilization of glucose and the shunting of metabolic precursors away from the oxidative function of the mitochondria mediated through the decreased expression of *Gdh*.

### Evidence of conserved mitochondrial retrograde signals are identified within the systems biologic analysis

Our previous genetic interaction studies had implicated p53 as being operative in the growth arrest associated with loss of *CoVa* (Mandal *et al.* 2005). CG17327 and CG2017 are two genes responsive to p53 (Akdemir *et al.* 2007) and are increased in response to *CoVa* RNAi (Figure 5). CG17327 is an aminoacyl- tRNA hydrolase, and CG2017 is a GTP binding protein acting as a possible protein synthesis factor. Interestingly, both of these genes were also identified in a screen for *Drosophila* oxidant stress employing hyperoxia (Gruenewald *et al.* 2009).

The gene *Augmenter of liver regeneration* (*Alr*), which contains both sulfhydryl oxidase and cytochrome c reductase activities (Thirunavukkarasu *et al.* 2008), is upregulated by *CoVa* RNAi. *Alr* maintains mitochondria in a rudimentary ultra-structure network that is associated with low oxidative capacity and is thought to be one mechanism that maintains pluripotency in a murine hematopoietic stem cell model (Todd *et al.* 2010; Wilkerson and Sankar 2011). In a separate model of mitochondrial dysfunction employing the *Drosophila* mutant *total knock out*, CG30022, a mitochondrial hydrolase, and Cyp6a23 were identified to be upregulated (Fernandez-Ayala *et al.* 2010). Both CG30022 and Cyp6a23 are found to be upregulated in response to *CoVa* RNAi and therefore are part of a conserved response to mitochondrial dysfunction (Figure 5).

Glutathione S-transferases (GST) carry out a wide range of cellular functions, including the removal of reactive oxygen species, regeneration of S-thiolated proteins and antioxidants (Dixon *et al.* 2011), and catalysis of the conjugation of reduced glutathione to both endogenous compounds as well as exogenous xenobiotics (Sheehan *et al.* 2001). *GstE9* is a cytosolic protein of the GST epsilon class previously described to be found in response to an oxidant stress (Li *et al.* 2008), as well as part of a larger transcriptional network associated with oxidative phosphorylation genes (Pile *et al.* 2003).

As *Impl3* is one of the consistently highest upregulated genes by *CoVa* RNAi, examination of the known literature on lactate dehydrogenase regulation, coupled with an analysis of the lactate dehydrogenase 5′ regulatory region, identifies *Hifα* to be an important arbiter of *Impl3* expression (Bruick and Mcknight 2001). A known function of *Hifα* is to sense the ambient levels of oxygen, the final electron acceptor of the electron transport chain in the mitochondria. In addition, it is known that *Hifα* controls the expression of both glycolytic and cytochrome oxidase genes (Semenza *et al.* 1994; Fukuda *et al.* 2007). *Hifα* therefore provides an attractive candidate for mitochondrial retrograde signaling, as it senses the primary substrate reduced by the mitochondria, controls glycolytic substrate flux via the transcriptional control of glycolysis, and controls the expression of the cytochrome oxidase genes. A plausible model would therefore incorporate *Hifα* as a central player in mitochondrial retrograde signaling, most especially in times of mitochondrial dysfunction (Figure 5). It has long been known that inhibition of complex IV through pharmacologic means results in upregulation of glycolytic genes (Simon 1953). Examination of our profiling data through coregulated gene network analysis reveals that most of the genes responding to *CoVa* RNAi are correlated to *Impl3*, which suggests *Hifα* is a central gene directing the transcriptional response. When the stringent gene list describing the response to *CoVa* knockdown (Figure 2) was examined for the Hifα binding sites within the 500 base pairs surrounding the transcriptional start site of the gene, 19 of the 22 genes possess at least one high similarity Hifα binding site (Table S3). Further, the microarray data reveal transcriptional upregulation of *Hifα* (Figure 3). This is interesting in that most literature regarding *Hifα* has focused on the post transcriptional stabilization of *Hifα*, usually through the action of hydroxy-prolyl hydroxylase (Bruick and Mcknight 2001). Adding more complexity to the regulation of *Hif1α* is the recent report that *Hif1α* may be stabilized by alternative methods such as neddylation (Ryu *et al.* 2011), a mechanism of stabilization that requires the presence of reactive oxygen species. Traditionally the stabilization of *Hif1a* has been thought to require reactive oxygen species (Chandel *et al.* 2000); however, *Hif1α* may be stabilized in a reactive oxygen species–independent fashion (Chua *et al.* 2010). As our prior observation has revealed low reactive oxygen species generation with *CoVa* RNAi *in vivo*, we concur that stabilization of *Hif1α* may occur in the absence of reactive oxygen species. Undoubtedly, the regulation of *Hif1α* action is achieved through multiple mechanisms and will not be the same under all biologic conditions. Regardless of the specific mechanism by which *Hifα* mRNA is increased in response to *CoVa* knockdown, as glycolytic genes are found to be upregulated and Hifα binding sites are identified in the majority of the top differentially regulated genes, we conclude that *Hifα* is likely to play a key role in mitochondrial retrograde signaling in times of mitochondrial dysfunction.

We conclude that the most likely mitochondrial retrograde signals identified in our expression profiling experiments are *p53*, *alr*, CG30022, *Cyp6a23*, *GstE9*, and *Hifα* (Figure 5). These identified genes may be the mitochondrial retrograde signal itself or an output from a retrograde signal system. As these genes have been identified in other biologic systems, their importance in mitochondrial regulation of the nucleus must be emphasized. Further directed studies will clarify the roles that these genes play in modulating nuclear function.

### Cytochrome p450 proteins, developmental cues, nuclear pores, and redox generators are identifiable within the systems biologic analysis

Cytochrome p450 (CYP450) proteins comprise a large group of heme-containing proteins that predominantly perform oxidation reactions. Targets of CYP450 proteins include both endogenous molecules, such as steroid hormones and lipids, as well as exogenous compounds, such as xenobiotics. A transcriptional response that directs detoxifying xenobiotics may be a conserved reaction to counter the threat posed by xenobiotics, compounds that often have the electron transport chain as their target. The expressions of *Cyp4e2* and *Cyp9c1* are increased in response to *CoVa* RNAi, and interestingly, both of these proteins have been identified in a screen for class O *Forkhead box* protein function (Junger *et al.* 2003) and are thought to be increased in response to decreased insulin signaling (Figure 5).

Ecdysone is a steroidal prohormone that is involved in larval maturation and directs stage-dependent gene expression. *Jhl-26* (Dubrovsky *et al.* 2000; Palli 2009) and CG3714 (Beckstead *et al.* 2005) are known to be responsive to ecdysone and are both increased in response to *CoVa* knockdown. These two genes may therefore serve as potentially important links between energy status and developmental stage (Figure 5).

The nuclear pore is a large specialized multiprotein channel that connects the cytoplasm to the nucleus and is composed of nucleoporins. Nucleoporin 50 kD (Nup50) acts as a cofactor for the importin-alpha and importin-beta heterodimer, which allows for transportation of nuclear-targeted proteins through the nuclear pore complex (Lindsay *et al.* 2002). An example of a signaling molecule dependent upon nuclear pores and the importin-alpha and importin-beta system is the mothers against decapentaplegic class of protein of the transforming growth factor beta signaling cascade. Therefore, signaling molecules may be excluded from the nucleus as a direct result of cellular energy stores (Figure 5).

The last two genes identified in the stringent analysis include *thiamine pyrophosphate carrier protein 1* (*Tpc1*), a mitochondrial thiamine transporter (Iacopetta *et al.* 2010), and CG4829, a protein-glutamine-gamma glutamyl transferase, genes that are upregulated and downregulated, respectively, in response to *CoVa* RNAi (Figure 5). Gamma glutamyl transferases catalyze the transfer of glutamyl groups from reduced glutathione to water, proteins, and amino acids. In the process of this catalysis, hydrogen peroxide may be released, and thus, gamma glutamyl transferases have been shown to be a significant source of cellular oxidants (Maellaro *et al.* 2000).

## Summary

To conclude, transcriptional profiling of *CoVa* RNAi reveals the plasticity of the *Drosophila* cell to respond to experimental manipulation of the electron transport chain, and it provides molecular mechanisms for understanding how energy substrate management is controlled in times of stress. Through these studies, we witness the response of the transcriptional profile to experimentally induced electron transport deficiency and are able to discern retrograde mitochondrial signals and outputs. Understanding the mechanism through which the mitochondrion orchestrates a nuclear response and is able to manage the disposition of energy precursors may help to develop novel therapeutics for cancer, as well as to better understand the metabolic program of rapidly dividing cells that preferentially utilize glucose as their fuel source.

## Supplementary Material

Supporting Information
