# Supplementary material for: Expression Profiling of Attenuated Mitochondrial Function Identifies Retrograde Signals in Drosophila
Source: G3 (Bethesda). 2012 Aug 1;2(8):843–51. doi: 10.1534/g3.112.002584 (PMC3411240; doi:10.1534/g3.112.002584)
Supplement: Supporting Information [file supp_2_8_843__index.html]

Supporting Information 

# Expression Profiling of Attenuated Mitochondrial Function Identifies Retrograde Signals in *Drosophila*

## Supporting Information for Freije, Mandal, and Banerjee, 2012

**Files in this Data Supplement:**

- Supporting Information - Figure S1 and Tables S1-S3 (PDF, 108 KB)
- Figure S1 - CoVa transcripts are knocked-down after RNAi treatment (PDF, 51 KB)
- Table S1 - Affymetrix probesets differentially expressed in Drosophila S2 cells treated with *CoVa* RNAi using the criteria of a minimum of 1.5 times or greater difference within a 90% confidence bound; absolute difference greater than 200; and with a p-value less than 0.05 using a Welch modified two sample t-test (as compared to GFP controls) (PDF, 89 KB)
- Table S2 - GFP to *CoVa* fold change (microarray) and RQ (qRT-PCR) of selected glycolytic genes 72 hours after *CoVa* RNAi (PDF, 56 KB)
- Table S3 - Location of Hif alpha binding sites within the 5' region of the most differentially expressed genes changed by *CoVa* RNA (PDF, 70 KB)
