# Supplementary material for: Expression Profiling of Attenuated Mitochondrial Function Identifies Retrograde Signals in Drosophila
Source: G3 (Bethesda). 2012 Aug 1;2(8):843–51. doi: 10.1534/g3.112.002584 (PMC3411240; doi:10.1534/g3.112.002584)
Supplement: Supporting Information [file supp_2.8.843_002584SI.pdf]

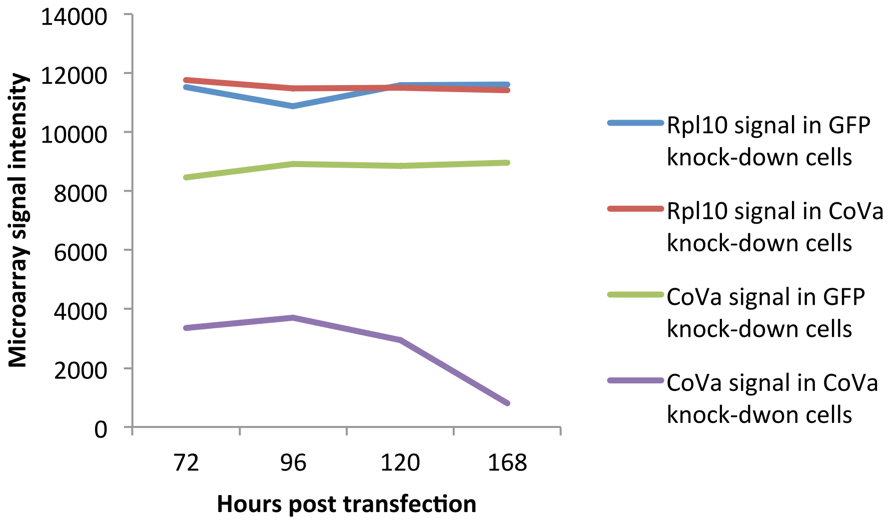

Figure S1 CoVa transcripts are knocked-down after RNAi treatment. Shown is the microarray signal intensity of Cova and Rpl10 over the time course of an independent experiment. Rpl10 serves as a control gene which remains invariant over the 168 hours of observation in both the GFP transfected controls and CoVa knock-down cells. CoVa signal intensity is markedly reduced at the first time point interrogated (72 hours; 60% decrease), and is further lowered by 91% at the termination of the experiment at 168 hours. The mean knock-down of CoVa expression at the last time-point across the three experiments is 70%.

Table S1 Affymetrix probesets differentially expressed in Drosophila S2 cells treated with *CoVa* RNAi using the criteria of a minimum of 1.5 times or greater difference within a 90% confidence bound; absolute difference greater than 200; and with a p-value less than 0.05 using a Welch modified two sample t-test (as compared to GFP controls).

Probe set	Gene	Fold change	Difference of means	p-value
1637772_at	CG4726	35.96	1717.76	0.022778
1628657_at	GstE9	28.72	1772.83	0.008169
1635227_at	ImpL3	16.22	3500.69	0.001307
1633039_at	CG5646	13	269.38	0.028285
1627073_a_at	CG10126	12.5	366.64	0.045699
1627582_a_at	CG30035	7.35	702.39	0.003059
1633536_at	CG4630	7.02	432.34	0.00703
1633639_at	Cyp28d1	6.9	207.98	0.035287
1624101_at	Cyp6a23	5.74	659.64	0.015528
1632802_at	JhI-26	4.98	1868.33	0.000944
1633238_at	GstE8	4.36	1365.31	0.001478
1634739_a_at	Pfk	4.35	839.72	0.001846
1632652_s_at	CG30022	4.34	1399.74	0.0068
1628558_at	CG30022	4.27	1525.58	0.006038
1625031_at	CG7841	3.94	1401.63	0.000208
1634382_at	CG31675	3.69	308.02	0.005811
1627273_at	CG12035	3.58	336.73	0.003008
1629040_at	CG3476	3.56	312.88	0.001011
1628345_at	Cyp6a9	3.38	283.54	0.017882
1635900_at	Thor	3.31	1608.51	0.016801
1624156_at	Ugt86Da	3.3	1066.05	0.007207
1638562_a_at	Cyp6d5	3.21	254.12	0.021037
1638844_s_at	CG3714	3.2	1441.15	0.000687
1631620_at	GlyP	3.13	616.48	0.011416
1635684_a_at	unc-13	3.02	921.03	0.009756

1633599_a_at	Pepck	2.96	388.7	0.024467
1630885_at	CG12534	2.9	861.78	0.000345
1641428_at	Cyp9c1	2.89	797.79	0.005421
1635439_at	CG10063	2.87	222.44	0.035052
1639704_at	CG14695	2.79	209.18	0.010238
1632676_s_at	CG11897	2.76	992.05	0.010638
1627844_at	Cyp4e2	2.71	694.62	0.000188
1641606_s_at	CG6608	2.65	1132.26	0.001534
1633355_at	CG10802	2.64	964.05	0.000083
1634129_at	CG9663	2.58	361.56	0.007303
1624203_s_at	Gli	2.55	276.92	0.005201
1634152_at	GstD5	2.54	429.93	0.006045
1624982_s_at	CG5080	2.53	399.99	0.02619
1628660_at	CG7130	2.5	731.68	0.001244
1633771_s_at	CG2017	2.49	1044.97	0.000183
1626679_at	CG17327	2.47	1445.85	0.000145
1640489_at	CG18522	2.38	401.1	0.000308
1636305_a_at	CG17327	2.37	1238.84	0.000126
1628187_s_at	CG9691	2.34	1365.98	0.014949
1633803_at	Pgm	2.33	449.25	0.004743
1627939_a_at	ferrochelatase	2.33	1380.22	0.00052
1626653_a_at	ferrochelatase	2.33	1225.52	0.000596
1640341_s_at	Dgp-1	2.29	2067.66	0.000357
1628052_at	Cyp6a17	2.27	1661.54	0.004322
1624070_at	RpS9	2.27	627.65	0.002026
1640230_at	HDC05827	2.24	809.04	0.001293
1638216_at	CG13623	2.24	764.08	0.000793
1638693_s_at	CG1882	2.23	1681.48	0.000102

1623472_at	wus	2.2	573.49	0.000271
1634019_at	CG2064	2.14	1161.13	0.001623
1634930_at	CT35997	2.13	895.7	0.002344
1635619_a_at	cnn	2.11	1182.4	0.000058
1628328_at	GstE10	2.1	210.44	0.00546
1626460_at	CG2658	2.08	580.03	0.000279
1627945_at	Fdxh	2.05	705.04	0.001236
1640977_at	CG12264	2.04	1830.34	0.000264
1632958_a_at	CG15675	2.04	356.19	0.000435
1636174_at	GstD9	2.03	1547.22	0.002568
1628915_s_at	Exn	2.02	272.67	0.005396
1634528_at	CG8412	2.02	432.36	0.002476
1638852_at	CHKov2	2.02	249.1	0.003247
1630802_at	Cyp6d4	2.01	694.47	0.00191
1625026_at	CG3348	2.01	1318.8	0.006981
1623268_a_at	CG33785	2	1539.72	0.000073
1629039_at	asrij	1.99	755.41	0.000218
1639997_s_at	CT39116	1.98	1668.12	0.000015
1635547_a_at	Aats-trp	1.98	900.63	0.000203
1640185_at	CG2076	1.97	2376.19	0.000178
1640363_a_at	CG6330	1.96	1075.45	0.001794
1624029_at	CG3608	1.95	535.85	0.000331
1639621_at	CG10916	1.95	717.72	0.000491
1640884_at	CG15784	1.93	748.27	0.000161
1625162_at	CG4611	1.92	375.66	0.000005
1633622_at	CG3008	1.91	650.19	0.000014
1632707_at	CG12379	1.91	296.25	0.000408
1636289_s_at	DNApol-iota	1.9	304.62	0.001014

1641293_at	ire-1	1.9	367.76	0.002604
1636131_at	lig3	1.89	456.33	0.00038
1630975_at	CG2909	1.89	439.26	0.000137
1628683_at	CG6272	1.88	720.17	0.001066
1631704_at	CG5805	1.88	700.6	0.002559
1637816_s_at	CG2171-RA	1.88	2401.06	0.000529
1634374_at	CG33138	1.87	869.67	0.000279
1638511_at	Aats-trp	1.87	1460.39	0.000091
1629387_s_at	aru	1.86	843.94	0.000541
1634072_s_at	Hmgs	1.86	1819	0.000793
1640339_at	l(1)G0136	1.86	732.24	0.000698
1631822_at	mus205	1.84	262.68	0.000246
1634899_a_at	CG6512	1.83	1927.28	0.000012
1628099_at	bor	1.83	1949.76	0.000001
1634383_a_at	JhI-21	1.82	2017.67	0.000088
1627034_a_at	CG9410	1.8	423.06	0.000279
1637439_at	CG14709	1.79	1424.96	0.000059
1631688_at	mal	1.79	631.37	0.000106
1635848_at	Inos	1.77	1965.68	0.000019
1627343_a_at	CG5535	1.77	969.38	0.000169
1639142_s_at	CR_tc_GH14469	1.76	855.6	0.00003
1640075_a_at	path	1.76	1477.97	0.000134
1625496_at	Ide	1.74	1425.32	0.000248
1630857_s_at	NTPase	1.73	1913.7	0.000325
1632978_at	CG32207	1.73	640.8	0.000063
1641339_at	CG10137	1.72	252.02	0.000125
1627973_s_at	CG33075-RA	1.72	228.94	0.000214
1625763_at	CG2789	1.7	2771.2	0.000158

1638259_s_at	Aats-val	1.7	909.84	0.000043
1633956_s_at	CG7995	1.7	315.34	0.000052
1633641_a_at	CG15611	-1.8	-604.34	0.000001
1629889_s_at	regucalcin	-1.83	-1961.69	0.00004
1636311_at	Gpdh	-1.85	-541.06	0.000014
1625265_at	CG9119	-1.88	-281.64	0.000174
1631321_s_at	His1	-1.92	-1895.23	0.000033
1639962_a_at	*CoVa*	-1.95	-3949.65	0.00018
1639232_s_at	SP1029	-1.96	-459.18	0.000004
1628081_s_at	CG7530	-1.97	-974.41	0.000012
1629442_at	egr	-1.97	-572.29	0.000017
1629740_at	His1	-2.02	-303.53	0.000158
1625925_at	His2A	-2.02	-1272.88	0.000251
1632744_a_at	if	-2.07	-432	0.000018
1630150_s_at	Cg25C	-2.08	-210.65	0.000406
1629430_s_at	regucalcin	-2.09	-1136.35	0.00052
1637055_s_at	AC006215	-2.16	-512.22	0.000029
1626727_at	Mct1	-2.19	-232.73	0.000078
1630986_s_at	Adk3	-2.22	-200.35	0.002186
1640720_a_at	CG14872	-2.22	-456.3	0
1636835_at	CG16700	-2.41	-314.68	0.002155
1629572_a_at	fat-spondin	-2.44	-1833.55	0.000162
1625023_a_at	nAcRbeta-21C	-2.46	-570.14	0
1639766_at	Pgk	-2.75	-1913.94	0
1623885_at	alpha-Est1	-2.82	-246.05	0.002637
1641270_at	CG8745	-2.98	-321.44	0.001625
1623950_s_at	Ama	-3.05	-228	0.019889
1630141_at	CG2158	-3.19	-885.14	0

1638807_s_at	CG4829	-3.43	-532.51	0.000007
1637366_at	CG16876	-3.5	-379.47	0.000005
1640835_a_at	Gdh	-4.2	-2106.33	0
1635930_at	btn	-4.41	-237.06	0.000217
1634302_s_at	CG14516	-5.75	-298.51	0.000099

This genelist compared all GFP control samples to all *CoVa* RNAi samples and included all time points sampled of all three replicates. The fold change was calculated by dividing the mean *CoVa* microarray signal intensity and dividing by the mean GFP microarray intensity. The difference of means is the difference in mean microarray signal intensity between the two groups. The p-value is the result of a modified Welch two sample t-test. The probesets are ranked from highest up-regulated to highest down-regulated.

Table S2 GFP to *CoVa* fold change (microarray) and RQ (qRT-PCR) of selected glycolytic genes 72 hours after *CoVa* RNAi

Gene	Microarray*	RQ (SEM, p-value)#
Phosphofructokinase	1.6-2.4 ($p<0.002$)	3.0 (0.2, $p<0.0004$)
Phosphoglycerate kinase	0.3 ($p<1 \times 10^{-7}$)	0.3 (0.04, $p<0.0008$)
Ecdysone-inducible protein L3	4.7-5.9 ($p<0.001$)	3.3 (0.46, $p<0.009$)

RQ= relative quotient

*Two of the three microarray replicates were assayed at the 72 hour time point and the range of the ratios are displayed. The p-values were calculated by a Welch modified two sample t-test in the DCHIP program.

The standard error of the mean (SEM) of the corresponding GFP controls are 0.18, 0.07, and 0.147 for Pfk, Pgk, and Impl3 respectively. The p-values were calculated by Fisher's protected least significant difference.

Table S3 Location of Hifα binding sites within the 5' region of the most differentially expressed genes changed by *CoVa* RNAi.

Gene	Similarity score	Position	Sequence	Strand
CG4726	1	-430	GCGTG	+
GstE9	0.79	-353	AGGTG	-
ImpL3	0.82	-111	TCGTG	+
Cyp6a23	1	-238	ACGTG	+
JhI-26	0.82	-73	TCGTG	+
Pfk	0.79	-360	ATGTG	+
Pfk	0.79	-434	ATGTG	+
CG30022	0.82	-284	TCGTG	-
CG7841	1	-221	ACGTG	-
Ugt86Da	1	-402	ACGTG	+
CG3714	1	14	GCGTG	+
CG3714	1	-414	GCGTG	-
Alr	0.79	-403	GAGTG	-
Cyp9c1	1	-113	ACGTG	-
Cyp4e2	0.82	38	TCGTG	+
Tpc1	1	-398	GCGTG	-
Tpc1	1	-419	GCGTG	-
CG10802	1	41	ACGTG	+
CG2017	1	0	GCGTG	+
CG2017	1	-17	GCGTG	+
CG2017	1	-267	GCGTG	+
CG2017	1	-274	GCGTG	+
CG2017	1	-352	ACGTG	+
CG17327	0.82	-380	CCGTG	+
Pgk	0.82	-39	CCGTG	-
Nup50	1	-432	ACGTG	+

CG4829	1	45	GCGTG	-
CG4829	1	38	GCGTG	-
CG4829	1	-191	ACGTG	-
Gdh	1	-40	ACGTG	-
Gdh	1	-427	ACGTG	+
Gdh	1	-431	ACGTG	+

The 22 genes consistently and robustly altered by loss of *CoVa* expression (displayed in Figure 2) were examined for the consensus Hifα binding sequence of RCGTG (R is either A or G) within the area spanning from -450 to +50 of the transcription start site. The location of the Hifα binding site is displayed, as is the exact sequence found, the similarity score as compared to the consensus binding site, and whether the site is on the coding or complementary strand.
