# Supplementary material for: Expression Profiling of Attenuated Mitochondrial Function Identifies Retrograde Signals in Drosophila
Source: G3 (Bethesda). 2012 Aug 1;2(8):843–51. doi: 10.1534/g3.112.002584 (PMC3411240; doi:10.1534/g3.112.002584)
Supplement: Supporting Information [file supp_2.8.843_FigureS1.pdf]

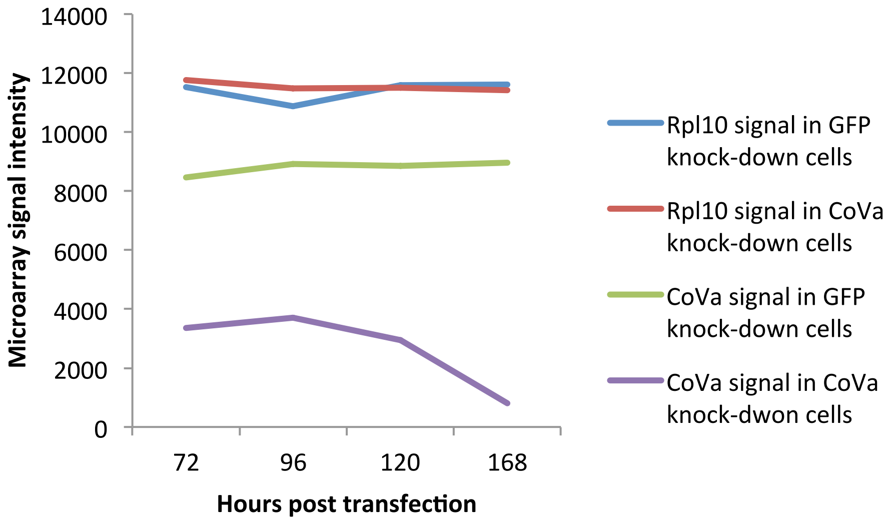

Figure S1 CoVa transcripts are knocked-down after RNAi treatment. Shown is the microarray signal intensity of Cova and Rpl10 over the time course of an independent experiment. Rpl10 serves as a control gene which remains invariant over the 168 hours of observation in both the GFP transfected controls and CoVa knock-down cells. CoVa signal intensity is markedly reduced at the first time point interrogated (72 hours; 60% decrease), and is further lowered by 91% at the termination of the experiment at 168 hours. The mean knock-down of CoVa expression at the last time-point across the three experiments is 70%.
