# Supplementary material for: Expression Profiling of Attenuated Mitochondrial Function Identifies Retrograde Signals in Drosophila
Source: G3 (Bethesda). 2012 Aug 1;2(8):843–51. doi: 10.1534/g3.112.002584 (PMC3411240; doi:10.1534/g3.112.002584)
Supplement: Supporting Information [file supp_2.8.843_TableS1.pdf]

Table S1 Affymetrix probesets differentially expressed in Drosophila S2 cells treated with *CoVa* RNAi using the criteria of a minimum of 1.5 times or greater difference within a 90% confidence bound; absolute difference greater than 200; and with a p-value less than 0.05 using a Welch modified two sample t-test (as compared to GFP controls).

Probe set	Gene	Fold change	Difference of means	p-value
1637772_at	CG4726	35.96	1717.76	0.022778
1628657_at	GstE9	28.72	1772.83	0.008169
1635227_at	ImpL3	16.22	3500.69	0.001307
1633039_at	CG5646	13	269.38	0.028285
1627073_a_at	CG10126	12.5	366.64	0.045699
1627582_a_at	CG30035	7.35	702.39	0.003059
1633536_at	CG4630	7.02	432.34	0.00703
1633639_at	Cyp28d1	6.9	207.98	0.035287
1624101_at	Cyp6a23	5.74	659.64	0.015528
1632802_at	JhI-26	4.98	1868.33	0.000944
1633238_at	GstE8	4.36	1365.31	0.001478
1634739_a_at	Pfk	4.35	839.72	0.001846
1632652_s_at	CG30022	4.34	1399.74	0.0068
1628558_at	CG30022	4.27	1525.58	0.006038
1625031_at	CG7841	3.94	1401.63	0.000208
1634382_at	CG31675	3.69	308.02	0.005811
1627273_at	CG12035	3.58	336.73	0.003008
1629040_at	CG3476	3.56	312.88	0.001011
1628345_at	Cyp6a9	3.38	283.54	0.017882
1635900_at	Thor	3.31	1608.51	0.016801
1624156_at	Ugt86Da	3.3	1066.05	0.007207
1638562_a_at	Cyp6d5	3.21	254.12	0.021037
1638844_s_at	CG3714	3.2	1441.15	0.000687
1631620_at	GlyP	3.13	616.48	0.011416
1635684_a_at	unc-13	3.02	921.03	0.009756

1633599_a_at	Pepck	2.96	388.7	0.024467
1630885_at	CG12534	2.9	861.78	0.000345
1641428_at	Cyp9c1	2.89	797.79	0.005421
1635439_at	CG10063	2.87	222.44	0.035052
1639704_at	CG14695	2.79	209.18	0.010238
1632676_s_at	CG11897	2.76	992.05	0.010638
1627844_at	Cyp4e2	2.71	694.62	0.000188
1641606_s_at	CG6608	2.65	1132.26	0.001534
1633355_at	CG10802	2.64	964.05	0.000083
1634129_at	CG9663	2.58	361.56	0.007303
1624203_s_at	Gli	2.55	276.92	0.005201
1634152_at	GstD5	2.54	429.93	0.006045
1624982_s_at	CG5080	2.53	399.99	0.02619
1628660_at	CG7130	2.5	731.68	0.001244
1633771_s_at	CG2017	2.49	1044.97	0.000183
1626679_at	CG17327	2.47	1445.85	0.000145
1640489_at	CG18522	2.38	401.1	0.000308
1636305_a_at	CG17327	2.37	1238.84	0.000126
1628187_s_at	CG9691	2.34	1365.98	0.014949
1633803_at	Pgm	2.33	449.25	0.004743
1627939_a_at	ferrochelatase	2.33	1380.22	0.00052
1626653_a_at	ferrochelatase	2.33	1225.52	0.000596
1640341_s_at	Dgp-1	2.29	2067.66	0.000357
1628052_at	Cyp6a17	2.27	1661.54	0.004322
1624070_at	RpS9	2.27	627.65	0.002026
1640230_at	HDC05827	2.24	809.04	0.001293
1638216_at	CG13623	2.24	764.08	0.000793
1638693_s_at	CG1882	2.23	1681.48	0.000102

1623472_at	wus	2.2	573.49	0.000271
1634019_at	CG2064	2.14	1161.13	0.001623
1634930_at	CT35997	2.13	895.7	0.002344
1635619_a_at	cnn	2.11	1182.4	0.000058
1628328_at	GstE10	2.1	210.44	0.00546
1626460_at	CG2658	2.08	580.03	0.000279
1627945_at	Fdxh	2.05	705.04	0.001236
1640977_at	CG12264	2.04	1830.34	0.000264
1632958_a_at	CG15675	2.04	356.19	0.000435
1636174_at	GstD9	2.03	1547.22	0.002568
1628915_s_at	Exn	2.02	272.67	0.005396
1634528_at	CG8412	2.02	432.36	0.002476
1638852_at	CHKov2	2.02	249.1	0.003247
1630802_at	Cyp6d4	2.01	694.47	0.00191
1625026_at	CG3348	2.01	1318.8	0.006981
1623268_a_at	CG33785	2	1539.72	0.000073
1629039_at	asrij	1.99	755.41	0.000218
1639997_s_at	CT39116	1.98	1668.12	0.000015
1635547_a_at	Aats-trp	1.98	900.63	0.000203
1640185_at	CG2076	1.97	2376.19	0.000178
1640363_a_at	CG6330	1.96	1075.45	0.001794
1624029_at	CG3608	1.95	535.85	0.000331
1639621_at	CG10916	1.95	717.72	0.000491
1640884_at	CG15784	1.93	748.27	0.000161
1625162_at	CG4611	1.92	375.66	0.000005
1633622_at	CG3008	1.91	650.19	0.000014
1632707_at	CG12379	1.91	296.25	0.000408
1636289_s_at	DNApol-iota	1.9	304.62	0.001014

1641293_at	ire-1	1.9	367.76	0.002604
1636131_at	lig3	1.89	456.33	0.00038
1630975_at	CG2909	1.89	439.26	0.000137
1628683_at	CG6272	1.88	720.17	0.001066
1631704_at	CG5805	1.88	700.6	0.002559
1637816_s_at	CG2171-RA	1.88	2401.06	0.000529
1634374_at	CG33138	1.87	869.67	0.000279
1638511_at	Aats-trp	1.87	1460.39	0.000091
1629387_s_at	aru	1.86	843.94	0.000541
1634072_s_at	Hmgs	1.86	1819	0.000793
1640339_at	l(1)G0136	1.86	732.24	0.000698
1631822_at	mus205	1.84	262.68	0.000246
1634899_a_at	CG6512	1.83	1927.28	0.000012
1628099_at	bor	1.83	1949.76	0.000001
1634383_a_at	JhI-21	1.82	2017.67	0.000088
1627034_a_at	CG9410	1.8	423.06	0.000279
1637439_at	CG14709	1.79	1424.96	0.000059
1631688_at	mal	1.79	631.37	0.000106
1635848_at	Inos	1.77	1965.68	0.000019
1627343_a_at	CG5535	1.77	969.38	0.000169
1639142_s_at	CR_tc_GH14469	1.76	855.6	0.00003
1640075_a_at	path	1.76	1477.97	0.000134
1625496_at	Ide	1.74	1425.32	0.000248
1630857_s_at	NTPase	1.73	1913.7	0.000325
1632978_at	CG32207	1.73	640.8	0.000063
1641339_at	CG10137	1.72	252.02	0.000125
1627973_s_at	CG33075-RA	1.72	228.94	0.000214
1625763_at	CG2789	1.7	2771.2	0.000158

1638259_s_at	Aats-val	1.7	909.84	0.000043
1633956_s_at	CG7995	1.7	315.34	0.000052
1633641_a_at	CG15611	-1.8	-604.34	0.000001
1629889_s_at	regucalcin	-1.83	-1961.69	0.00004
1636311_at	Gpdh	-1.85	-541.06	0.000014
1625265_at	CG9119	-1.88	-281.64	0.000174
1631321_s_at	His1	-1.92	-1895.23	0.000033
1639962_a_at	*CoVa*	-1.95	-3949.65	0.00018
1639232_s_at	SP1029	-1.96	-459.18	0.000004
1628081_s_at	CG7530	-1.97	-974.41	0.000012
1629442_at	egr	-1.97	-572.29	0.000017
1629740_at	His1	-2.02	-303.53	0.000158
1625925_at	His2A	-2.02	-1272.88	0.000251
1632744_a_at	if	-2.07	-432	0.000018
1630150_s_at	Cg25C	-2.08	-210.65	0.000406
1629430_s_at	regucalcin	-2.09	-1136.35	0.00052
1637055_s_at	AC006215	-2.16	-512.22	0.000029
1626727_at	Mct1	-2.19	-232.73	0.000078
1630986_s_at	Adk3	-2.22	-200.35	0.002186
1640720_a_at	CG14872	-2.22	-456.3	0
1636835_at	CG16700	-2.41	-314.68	0.002155
1629572_a_at	fat-spondin	-2.44	-1833.55	0.000162
1625023_a_at	nAcRbeta-21C	-2.46	-570.14	0
1639766_at	Pgk	-2.75	-1913.94	0
1623885_at	alpha-Est1	-2.82	-246.05	0.002637
1641270_at	CG8745	-2.98	-321.44	0.001625
1623950_s_at	Ama	-3.05	-228	0.019889
1630141_at	CG2158	-3.19	-885.14	0

1638807_s_at	CG4829	-3.43	-532.51	0.000007
1637366_at	CG16876	-3.5	-379.47	0.000005
1640835_a_at	Gdh	-4.2	-2106.33	0
1635930_at	btn	-4.41	-237.06	0.000217
1634302_s_at	CG14516	-5.75	-298.51	0.000099

This genelist compared all GFP control samples to all *CoVa* RNAi samples and included all time points sampled of all three replicates. The fold change was calculated by dividing the mean *CoVa* microarray signal intensity and dividing by the mean GFP microarray intensity. The difference of means is the difference in mean microarray signal intensity between the two groups. The p-value is the result of a modified Welch two sample t-test. The probesets are ranked from highest up-regulated to highest down-regulated.
