# Supplementary material for: Expression Profiling of Attenuated Mitochondrial Function Identifies Retrograde Signals in Drosophila
Source: G3 (Bethesda). 2012 Aug 1;2(8):843–51. doi: 10.1534/g3.112.002584 (PMC3411240; doi:10.1534/g3.112.002584)
Supplement: Supporting Information [file supp_2.8.843_TableS2.pdf]

Table S2 GFP to *CoVa* fold change (microarray) and RQ (qRT-PCR) of selected glycolytic genes 72 hours after *CoVa* RNAi

Gene	Microarray*	RQ (SEM, p-value)#
Phosphofructokinase	1.6-2.4 ($p<0.002$)	3.0 (0.2, $p<0.0004$)
Phosphoglycerate kinase	0.3 ($p<1 \times 10^{-7}$)	0.3 (0.04, $p<0.0008$)
Ecdysone-inducible protein L3	4.7-5.9 ($p<0.001$)	3.3 (0.46, $p<0.009$)

RQ= relative quotient

*Two of the three microarray replicates were assayed at the 72 hour time point and the range of the ratios are displayed. The p-values were calculated by a Welch modified two sample t-test in the DCHIP program.

The standard error of the mean (SEM) of the corresponding GFP controls are 0.18, 0.07, and 0.147 for Pfk, Pgk, and Impl3 respectively. The p-values were calculated by Fisher's protected least significant difference.
