# Supplementary material for: Expression Profiling of Attenuated Mitochondrial Function Identifies Retrograde Signals in Drosophila
Source: G3 (Bethesda). 2012 Aug 1;2(8):843–51. doi: 10.1534/g3.112.002584 (PMC3411240; doi:10.1534/g3.112.002584)
Supplement: Supporting Information [file supp_2.8.843_TableS3.pdf]

Table S3 Location of Hifα binding sites within the 5' region of the most differentially expressed genes changed by *CoVa* RNAi.

Gene	Similarity score	Position	Sequence	Strand
CG4726	1	-430	GCGTG	+
GstE9	0.79	-353	AGGTG	-
ImpL3	0.82	-111	TCGTG	+
Cyp6a23	1	-238	ACGTG	+
JhI-26	0.82	-73	TCGTG	+
Pfk	0.79	-360	ATGTG	+
Pfk	0.79	-434	ATGTG	+
CG30022	0.82	-284	TCGTG	-
CG7841	1	-221	ACGTG	-
Ugt86Da	1	-402	ACGTG	+
CG3714	1	14	GCGTG	+
CG3714	1	-414	GCGTG	-
Alr	0.79	-403	GAGTG	-
Cyp9c1	1	-113	ACGTG	-
Cyp4e2	0.82	38	TCGTG	+
Tpc1	1	-398	GCGTG	-
Tpc1	1	-419	GCGTG	-
CG10802	1	41	ACGTG	+
CG2017	1	0	GCGTG	+
CG2017	1	-17	GCGTG	+
CG2017	1	-267	GCGTG	+
CG2017	1	-274	GCGTG	+
CG2017	1	-352	ACGTG	+
CG17327	0.82	-380	CCGTG	+
Pgk	0.82	-39	CCGTG	-
Nup50	1	-432	ACGTG	+

CG4829	1	45	GCGTG	-
CG4829	1	38	GCGTG	-
CG4829	1	-191	ACGTG	-
Gdh	1	-40	ACGTG	-
Gdh	1	-427	ACGTG	+
Gdh	1	-431	ACGTG	+

The 22 genes consistently and robustly altered by loss of *CoVa* expression (displayed in Figure 2) were examined for the consensus Hifα binding sequence of RCGTG (R is either A or G) within the area spanning from -450 to +50 of the transcription start site. The location of the Hifα binding site is displayed, as is the exact sequence found, the similarity score as compared to the consensus binding site, and whether the site is on the coding or complementary strand.
